# Matchmaker of Resources, Influencers of Change: Ohio Mobility Managers in Action

**DOI:** 10.1093/geroni/igaf122.1692

**Published:** 2025-12-31

**Authors:** Holly Dabelko-Schoeny, Phoebe Allebach

**Affiliations:** The Ohio State University, Columbus, Ohio, United States; The Ohio State University, Columbus, Ohio, United States

## Abstract

Few alternative transportation (AT) options exist in many communities, and older adults have low rates of alternative transportation utilization, which is associated with higher levels of social isolation and depression. Developing and sustaining responsive AT options for older adults is a crucial step in the creation of age-friendly communities. Mobility managers work to improve transportation access for residents in their service areas and may play an important role in supporting AT development. Through a statewide survey of Ohio’s mobility managers (N = 39), we explored the role mobility managers play in supporting AT use and the infrastructure challenges and opportunities they perceive in AT utilization. Mobility managers reported that nearly half (41%) had not worked in transportation before becoming a mobility manager, and 48% did not receive any training on mobility management before starting their positions. Respondents believe their most important job responsibilities were leading advisory groups (63%), attending community meetings to advocate for transportation options (59%), and providing training to individual residents about transportation options (37%). Despite advocating for improved services, respondents perceived significant service barriers to AT use. These included infrequent service and limited hours (52%), insufficient service coverage area (48%), rides needed outside of the county (41%), and transportation services at capacity (41%). The findings highlight policy and practice implications for how AT options and utilization could be improved in Ohio, such as standardized education and training for mobility managers, regional-level transportation planning, and collaboration with local organizations (e.g., area agencies on aging).

